# Massive Microbiological Groundwater Contamination Associated with a Waterborne Outbreak in Lake Erie, South Bass Island, Ohio

**DOI:** 10.1289/ehp.9430

**Published:** 2007-02-06

**Authors:** Theng-Theng Fong, Linda S. Mansfield, David L. Wilson, David J. Schwab, Stephanie L. Molloy, Joan B. Rose

**Affiliations:** 1 Department of Crop and Soil Science; 2 Department of Microbiology and Molecular Genetics; 3 National Food Safety and Toxicology Center and; 4 Department of Food Science and Human Nutrition, Michigan State University, East Lansing, Michigan, USA; 5 National Oceanic and Atmospheric Administration Great Lake Environmental Research Laboratory, Ann Arbor, Michigan, USA; 6 Department of Fisheries and Wildlife, Michigan State University, East Lansing, Michigan, USA

**Keywords:** *Arcobacter*, groundwater, microbiological contamination, outbreak, viruses, waterborne

## Abstract

**Background:**

A groundwater-associated outbreak affected approximately 1,450 residents and visitors of South Bass Island, Ohio, between July and September 2004.

**Objectives:**

To examine the microbiological quality of groundwater wells located on South Bass Island, we sampled 16 wells that provide potable water to public water systems 15–21 September 2004.

**Methods:**

We tested groundwater wells for fecal indicators, enteric viruses and bacteria, and protozoa (*Cryptosporidium* and *Giardia*). The hydrodynamics of Lake Erie were examined to explore the possible surface water–groundwater interactions.

**Results:**

All wells were positive for both total coliform and *Escherichia coli*. Seven wells tested positive for enterococci and *Arcobacter* (an emerging bacterial pathogen), and F^+^-specific coliphage was present in four wells. Three wells were positive for all three bacterial indicators, coliphages, and *Arcobacter*; adenovirus DNA was recovered from two of these wells. We found a cluster of the most contaminated wells at the southeast side of the island.

**Conclusions:**

Massive groundwater contamination on the island was likely caused by transport of microbiological contaminants from wastewater treatment facilities and septic tanks to the lake and the subsurface, after extreme precipitation events in May–July 2004. This likely raised the water table, saturated the subsurface, and along with very strong Lake Erie currents on 24 July, forced a surge in water levels and rapid surface water–groundwater interchange throughout the island. Landsat images showed massive influx of organic material and turbidity surrounding the island before the peak of the outbreak. These combinations of factors and information can be used to examine vulnerabilities in other coastal systems. Both wastewater and drinking water issues are now being addressed by the Ohio Environmental Protection Agency and the Ohio Department of Health.

Contaminated groundwater is the most commonly reported source of waterborne disease in the United States, associated with 64% of the drinking water outbreaks between 1989 and 2002. In recent national figures (2001–2002), groundwater sources constituted 92% of the outbreaks, which often occurred in small communities (Blackburn et al. 2004). A large groundwater-associated outbreak in the Great Lakes basin occurred between June and September 2004 on South Bass Island, Ohio, affecting approximately 1,450 individuals (both residents and visitors) [Ohio Environmental Protection Agency (EPA) 2005]. The present study was undertaken to investigate the groundwater quality on the island and the factors associated with the contamination event.

South Bass Island is located in Ottawa County, Ohio, off the southern coast of Lake Erie, and approximately 5 mi from the Canadian border (Figure 1). South Bass Island is one of the main tourist destinations in the Midwest and has the nickname “Key West of the Midwest.” Most bars and restaurants on the island are located in the village of Put-in-Bay, which is the largest community on the island; Put-in-Bay has a permanent population of 350 and up to 25,000 visitors/day during the tourist season. Potable water on the island is provided through a number of public and private water systems. A public water system was defined by the Ohio EPA (2005) as a system that has at least 15 service connections or regularly serves an average of at least 25 individuals daily at least 60 days of the year.

The village of Put-in-Bay is served by a municipal public water system that uses primarily treated surface water from Lake Erie. However, many businesses and the majority of residents on the island use untreated groundwater pumped from wells on their premises as their primary source of potable water. There are approximately 13 transient noncommunity public water systems and small businesses on the island that use wells to meet their water needs. According to the Ohio EPA (2005), transient noncommunity public water systems are water systems that do not regularly serve at least 25 of the same persons over 6 months of the year (e.g., restaurants, campgrounds, gas stations).

During the time of the outbreak, South Bass Island used three main types of waste-water disposal systems: *a*) the village of Put-in-Bay operated a publicly owned treatment works (POTWs) that served the village; *b*) some of the businesses on the island were served by small package wastewater treatment works with aeration; and *c*) on-site waste-water treatment works, such as septic tanks, mound systems, subsurface sand filters, and holding tanks served most of the unincorporated areas of the island. The small package (or semipublic) wastewater treatment plants (WWTPs) are privately owned facilities that are regulated the same as POTWs by the Ohio EPA. All POTWs on South Bass Island discharge treated effluent to Lake Erie and are regulated by the Ohio EPA under a National Pollutant Discharge Elimination System (NPDES) permit. Under Ohio NPDES permits (Ohio EPA 2005), sanitary sewage treatment systems are allowed to discharge with a daily fecal coliform bacteria limit of 2,000 colony-forming units (CFU)/100 mL with a design flow of < 5,000 gal/day if they do not discharge directly into an Ohio river.

## Description of the outbreak

On 2 August 2004, the Ottawa County Health Department (OCHD) in Ohio received several telephone calls from persons reporting gastrointestinal illness after visiting South Bass Island. A food-borne disease outbreak investigation was initiated by the OCHD and the Ohio Department of Health (ODH). On 12 August 2004 the Ohio EPA was informed about a possible waterborne outbreak and began an investigation of the drinking and wastewater systems. On 16 August 2004 the ODH reported to the Foodborne and Diarrheal Diseases Branch at the Centers for Disease Control and Prevention (CDC; Atlanta, GA) that there were 70 cases of gastroenteritis with illness onsets between 1 June and 16 August 2004, including two confirmed cases of campylobacteriosis. All cases had a common history of visiting South Bass Island before contracting gastroenteritis. The number of cases reported per day peaked around 15 August 2004 when 75 cases were reported (O’Reilly et al. 2007). On 26 August 2004 the ODH and the OCHD advised island residents with private well water to boil their drinking water or use bottled water, and the Ohio EPA began ordering water-use advisories for public water systems with an indication of contamination.

**Figure 2:**
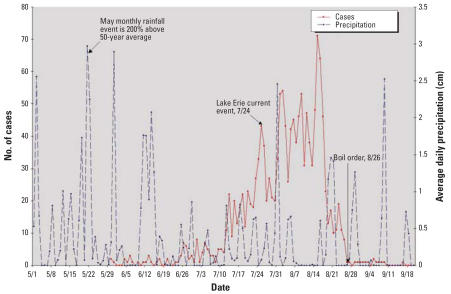
Gastroenteritis cases by date in 2004, average daily rainfall (cm), and key events over the duration of the outbreak. Index cases were reported on 30 May 2004 (*n* = 1,450); the estimated number of cases was obtained from O’Reilly et al. (2007).

By 5 September 2004, approximately 1,450 gastroenteritis cases had been reported. Figure 2 shows those cases based on the epidemiologic study undertaken (O’Reilly et al. 2007). The majority of cases occurred between 25 July and 17 August. The CDC detected a mixture of pathogens, including *Campylobacter* spp., Norovirus, *Giardia* spp., and *Salmonella typhimurium* in human fecal specimens (O’Reilly et al. 2007).

A case–control study was conducted by the CDC and the ODH between 30 August and 7 September 2004. A significant association between gastroenteritis symptoms and tap water consumption, as well as the amount of tap water consumed, on the island was reported both from wells and the municipal system (O’Reilly et al. 2007). Whereas recreational exposure (i.e., direct contact with the lake, swimming or wading in the lake water, swimming in any pool on the island) was not shown to be statistically associated with illness, direct exposure to Lake Erie did show an increased odds ratio (OR), with 13% of the cases and 8% of the controls showing illness [matched OR = 6.0; *p* = 0.1; 95% confidence interval (CI), 0.7–276] (O’Reilly 2005).

During 23–31 August 2004, the Ohio EPA and the CDC performed another study sampling for total coliform, *Escherichia coli,* and other enteric pathogens such as *Campylobactor* at locations identified during the previous epidemiologic investigation (O’Reilly et al. 2007). They also performed macroscopic particulate analysis from those wells. During 8–10 September 2004, the ODH conducted an extensive environmental assessment and water quality monitoring investigation of private wells on the island.

The present study was initiated to assist the Ohio EPA, following the CDC’s initial investigation, to further examine the extent of microbiological quality of the groundwater wells located on South Bass Island. Approximately 1 month after the peak of the outbreak, samples were collected from 16 wells that provide potable water to public and private water systems on the island in order to examine the ongoing risk to the population and residual distribution of the contamination on the island. Groundwater analyses included conventional indicators used for water, such as coliform bacteria (including total coliform and *E. coli*), and the alternative indicators enterococci, coliphage, and *Clostridium perfringens*. The wells were also tested for *Campylobacter*, *Giardia*, *Cryptosporidium*, and human enteric viruses. The U.S. EPA standards for drinking water for total coliform bacteria are < 1/100 mL (U.S. EPA 1986, 2001d). Other fecal indicators (*E. coli*, enterococci, and coliphage) and pathogens should not be present. In addition, we explored the factors associated with the microbial contaminants and their transport using climate and satellite data as well as a hydrodynamics model of Lake Erie.

## Materials and Methods

### Sampling sites

**Figure 3:**
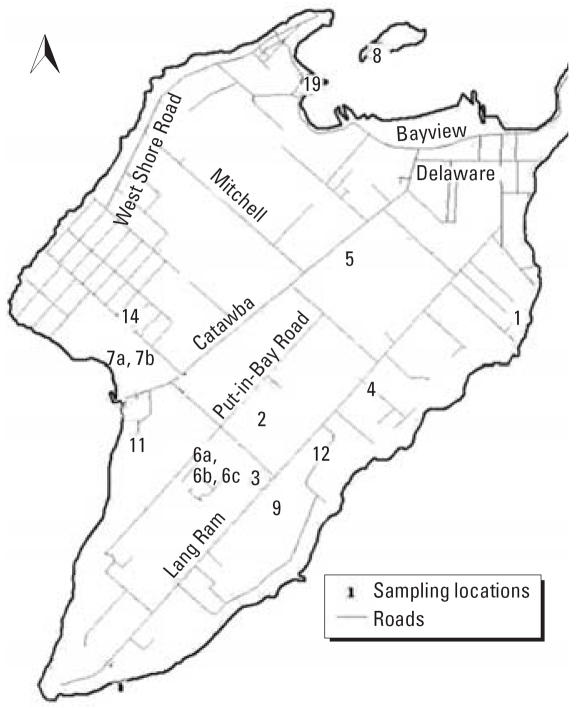
Wells sampled on South Bass Island 15–21 September 2004; map reproduced with permission from the Ohio EPA (2005).

A total of 16 wells at Put-in-Bay were selected by the Ohio EPA sampling team (15–21 September 2004) for study. Figure 3 shows the layout of the island and the sampling sites. The 16 wells were labeled according to the Ohio EPA labeling system (PB-1, PB-2, PB-3, PB-4, PB-5, PB-6A, PB-6B, PB-6C, PB-7A, PB-7B, PB-8, PB-9, PB-11, PB-12, PB-14, PB-19). Water samples were collected from various types of business including cottages/home rentals (*n* = 6), parks (*n* = 3), food services (*n* = 2), an apartment building, a public pool, a campground, a research facility, and a water treatment plant. Wells were not disinfected before the outbreaks but some were chlorinated after the outbreak. At 12 of the sites, semipublic facilities (package WWTPs) were used for waste-water treatment. Most of the unincorporated areas of South Bass Island are served by on-site wastewater treatment works. On-site systems typically consist of a septic tank and leach field. Two sites (PB-9 and PB-19) were served by on-site systems: PB-9 (airport) used a mounded system, and PB-19 [ODNR (Ohio Department of Natural Resources) Oak Point picnic area] used a subsurface sand filter. One site (PB-5; Horny Toad) was served by the village of Put-in-Bay full-scale publicly owned sewage treatment plant (Ohio EPA 2005). A septage disposal site was located in the middle of the island, between Catawba Rd. and Put-in-Bay Rd.

### Bacterial indicators and coliphage analyses

Grab samples were collected using sterile bottles; sodium thiosulfate was added to neutralize water samples with detectable chlorine residuals. Samples were processed within 24 hr of collection. Total coliform and *E. coli* were analyzed by a filtration/agar method (American Public Health Association et al. 1995) and by the Colilert Presence/Absence test kit (IDEXX Laboratories, Inc., Westbrook, ME). Aliquots of each water sample (300 mL) were filtered through a membrane filter and enumerated on mENDO (total coliform) media (catalog no. 273620) and EC-MUG (*E. coli*) media (catalog no. 222200), both from Difco Laboratories, (Detroit, MI). Enterococci were enumerated by membrane filtration and cultured on mEI agar (catalog no. 214885; Difco Laboratories) following U.S. EPA Method 1600 (U.S. EPA 2002). MCP medium (catalog no. 7477A; Acumedia, Baltimore, MD) was used for *C. perfringens* analysis (Bisson and Cabelli 1979). After incubation, yellow colonies that turned red or dark pink after being exposed to ammonium hydroxide were counted as *C. perfringens*. Each sample was analyzed in triplicate.

We analyzed coliphages using the double agar layer (DAL) method or enrichment method as described in U.S. EPA Methods 1601 and 1602 (U.S. EPA 2001a, 2001b). We used hosts *E. coli* F-amp (ATCC no. 700891; American Type Culture Collection, Manassas, VA) for detecting F^+^-specific coliphage and *E. coli* C3000 (ATCC no. 15597) for total (somatic and F^+^-specific) coliphages. In brief, we prefiltered 20 mL of each water sample through a 0.22-μm filter to remove debris and bacteria: 0.5 mL host and 2 mL filtered sample were then added to 3 mL trypticase soy broth (TSB) containing 1.5% agar before mixing and pouring onto a tryptic soy agar plate. Five replicates were assayed for a total of 10 mL. Overlays were incubated at 37°C for 24 hr and then assessed for plaque formation.

An enrichment step was performed on samples that were negative by DAL procedure to amplify the number of coliphage in the samples. A 1-L sample was supplemented with 12.5 mL 4 M MgCl_2_ and 5 mL host organism (*E. coli* C3000 or *E. coli* F amp) in log phase and 50 mL 10X TSB. For enrichment with *E. coli* C3000, we also added 10 mL/L 1% nalidixic acid solution. For *E. coli* F^+^amp as the host organism, enrichments were supplemented with 10 mL streptomycin/ampicillin solution. Enrichments were then incubated for 16–24 hr at 37°C. Phage presence in the enrichments was confirmed via plaque formation using the overlay method described above.

### Arcobacter/Campylobacter *spp. analysis.*

Four liters of grab samples were collected from each well initially for analysis of *Campylobacter* spp. Concentrated Maximum Recovery Diluent (Oxoid, Basingstoke, UK) was then added to each water sample at a ratio of 1:10. Samples were filtered through 0.45-μm membrane filters (Gelman Sciences, Ann Arbor, MI). After filtration, the membranes were then placed on Bolton Selective Enrichment Agar (BSEA; Oxoid) and flooded with 10 mL Bolton broth supplemented with 5% defibrinated sheep’s blood, cefoperazone (20 μg/mL), vancomycin (10 μg/mL), and amphotericin B (2 μg/mL). The plates (and all other incubations) were placed in an anaerobic jar and incubated at 37°C with 40 rpm agitation under an atmosphere of 10% CO_2_, 10% H_2_, and 80% N_2_. After 48 hr incubation, turbid broth cultures were diluted serially, plated onto BSEA, and incubated as above without agitation. Five isolated colonies from the enrichment cultures that produced growth were passaged onto BSEA to produce pure culture.

We performed *C. jejuni*–specific polymerase chain reaction (PCR) analysis to rapidly screen colonies isolated on BSEA (Wilson et al. 2000). Colonies were transferred by sterile toothpicks to PuReTaq Ready-To-Go PCR beads (Amersham Biosciences, Bucks, UK). PCR was performed with primer concentrations at 1 pmol/μL. Chromosomal DNA was extracted with Easy DNA (Invitrogen, Carlsbad, CA) from selected BSEA isolates. PCR-restriction fragment length polymorphism (PCR-RFLP) was then used to distinguish between *Campylobacter*, *Helicobacter,* and *Arcobacter* spp. (Marshall et al. 1999). This PCR amplifies a 1,004-bp fragment within the coding region of the 16S rRNA gene in *Campylobacter*, *Arcobacter*, and *Helicobacter* spp. Thereafter, all of these genera can be identified to the genus level with one restriction enzyme.

### Cryptosporidium *spp. and* Giardia *spp. analysis.*

For parasite analysis (*Cryptosporidium* spp. and *Giardia* spp.), approximately 100 L water was filtered through an Envirochek HV filter (Pall Gelman Laboratories, Ann Arbor, MI) at each sampling site. Filtration, elution, and recovery of parasites from filters were performed following U.S. EPA method 1623 (U.S. EPA 2001c). Parasite oocysts/cysts were then concentrated into a pellet by the Dynal Immunomagnetic Separation Technique (IMS; Dynabeads CG-combo Kit; Dynal Biotech, Inc., Lake Success, NY). The concentrated pellet was resuspended with MilliQ water [obtained from a Nanopure Diamond Analytical Ultrapure water system (Barnstead International, Dubuque, IA)] into 20 mL and split into two. One portion was screened for the presence of *Cryptosporidium* oocysts and *Giardia* cysts under Carl Zeiss Axioskop 2 fluorescence microscope (Zeiss, Thornwood, NY) after staining with monoclonal antibodies (EasyStain; Biotechnology Frontiers, North Ryde, Australia) tagged with fluorescein isothiocyanate. The pellet was also stained with a 0.4-μg/mL 4′6-diamidino-2-phenyl indole (DAPI) solution. Before staining, IMS concentrates were applied to Dynal Spot-On slides (Dynal Biotech, Oslo, Norway) and air dried. After air-drying and fixing in methanol, all samples were stained with 50 μL EasyStain, followed by 1 mL DAPI and were examined by fluorescence microscopy. The remaining pellet suspensions were stored for further analysis [i.e., cell culture if a (oo)cyst was detected by microscopy].

### Human enteric virus analyses

Approximately 1,000 L of water at each site was filtered through a 1 MDS cartridge filter (CUNO Inc., Meriden, CT, USA). Virus elution and concentration was carried out by organic flocculation as described by Fout et al. (1996). Viruses were desorbed from the filters by two rounds of reverse passage of 1 L 1.5% beef extract solution (1.5% wt/vol beef extract, 0.05 M glycine, pH 9.0–9.5). Viruses were flocculated by the addition of ferric (III) chloride to a final concentration of 2.5 mM and by lowering the pH to 3.5 (Payment et al. 1984). Viral concentrates were centrifuged at 2,500 × *g* for 15 min, and the pellet was resuspended in 30 mL of 0.15 M sodium phosphate (final pH 9.0). Viruses were purified by centrifugation at 10,000 × *g* for 10 min; brought to a neutral pH; supplemented with 100 U penicillin, 100 μg streptomycin, and 0.25 μg amphotericin B; and stored at −80°C until analysis.

Culturable viruses were assayed on Buffalo Green Monkey cells with Eagle minimum essential medium supplemented with 2% fetal bovine serum and incubated at 36.5 ± 1°C for 14 days. Samples were examined daily for the development of cytopathic effects. All samples underwent a secondary passage after freeze-thaw. We used U.S. EPA MPN (Most Probable Number) software (U.S. EPA, Cincinnati, OH) to calculate the MPN/100 L values and confidence limits for viruses.

Enteric viruses were also detected by PCR. Concentrated water samples (6 mL) were further purified, concentrated, and desalted with Centriprep YM-50 concentrator columns (Millipore). The final volume of concentrated eluate recovered was approximately 750 μL. Concentrates were stored at −80°C until analysis. We extracted and purified viral RNA from concentrates using a QIAamp Viral RNA Mini Kit (Qiagen, Valencia, CA) following the manufacturer’s protocol. Purified viral RNA was eluted in 60 μL of RNase-free water. Each concentrated and purified sample was serially diluted to a concentration of 10^−1^ and stored at −20°C. Both concentrations were assayed to examine inhibition. We performed PCR amplification for norovirus (NV), human enterovirus (HEntV), and human adenovirus (HAdV) using the primer sets shown in Table 1. HEntV primer sets were able to detect at least 25 different HEntVs; echovirus 22 was not detected. We used a nested primer set designed by Allard et al. (1992) to amplify HAdV. The primers were able to identify 47 HAdV serotypes, including the more common HAdV types 2, 40, and 41 (Allard et al. 1992; Puig et al. 1994). Reverse transcriptase (RT)-PCR for noroviruses and enteroviruses was performed using GeneAmp Gold RNA PCR Core Kit (Applied Biosystems, Foster City, CA) according to manufacturer’s recommendations (Le Guyader et al. 1996). Primers NVp110 and NVp36 were used. Noroviruses G1 and G2 were used as positive controls (courteously provided by J. Massey, Michigan Department of Community Health, Lansing, MI). We performed RT-nested-PCR for HEntV following a modified protocol by Fong et al. (2005). The final amplicon size was 154 bp. Poliovirus 1, LSc strain (ATCC no. VR-59) was used as a positive control; MilliQ water was used as a negative control for all PCR assays.

### Lake Erie hydrodynamic modeling

We used a coastal ocean circulation model (Princeton Ocean Model; Blumberg and Mellor 1987) to simulate the currents in Lake Erie during 2004. The model uses observed winds from weather stations around the lakes and buoys in the lake to estimate currents and thermal structure on a horizontal grid with 2-km grid spacing. This model has been used extensively in the Great Lakes for operational coastal forecasting (Kelley et al. 1998; Schwab and Bedford 1999) and has generally proven to be quite accurate (Beletsky and Schwab 2001; Beletsky et al. 2003). Storm surges in Lake Erie have previously been shown to influence water levels (O’Connor et al. 1999). Currents were calculated on an hourly basis and show considerable hour-to-hour and day-to-day variability, mainly due to variability in the wind field. As an example of current variability around the South Bass Island area, we developed and examined plots of daily averaged, vertically integrated currents for 8 days during the outbreak period in 2004. Average monthly precipitation data for 2004 was obtained from the ODH (2005). True-color LandSat 7 images of western Lake Erie were obtained from OhioLINK (2004) during the period of suspected contamination.

## Results

### Groundwater quality

Four water samples were collected on 15, 16, 20, and 21 September 2004 for a total of 16 samples. The dates and the physical and chemical analyses of ground-water samples, including free Cl_2_, total Cl_2_, pH, turbidity, and total dissolved solids (TDS), are shown in Table 2. Four samples (PB-3, PB-4, PB-11, and PB-14) had free Cl_2_ and total Cl_2_ ranging from 0.08 to 0.49 mg/L and from 0.04 to 1.00 mg/L, respectively. The pH was fairly consistent, ranging from 6.8 to 7.4. High levels of turbidity were found in PB-8, PB-9, and PB-19, with turbidity of 6.5, 4.1, and 3.9 nephelometric turbidity units (NTU), respectively. The National Primary Drinking Water Regulations require a maximum turbidity level of < 1 NTU (U.S. EPA 1975). Ten of 16 wells (PB-1, PB-3, PB-4, PB-5, PB-6B, PB-6C, PB-7B, PB-8, PB-11, and PB-19) were found to have TDS levels that exceeded the National Secondary Drinking Water Regulations of 500 mg/L (U.S. EPA 1991).

Table 3 shows the results of the microbiological analyses arranged from the most contaminated wells to the least contaminated. Total coliforms, *E. coli*, enterococci, *C. perfringens*, total coliphages, and F^±^-specific coliphages were monitored as evidence of fecal contamination. Eleven wells were positive for total coliforms (range, 2.2–90 CFU/100 mL; mean, 12.55 CFU/100 mL) and eight wells were positive for *E. coli* (range, 0.1–4 CFU/100 mL; mean, 0.64 CFU/100 mL) by the membrane filtration method. All wells were positive for both total coliform and *E. coli* by the Colilert Presence/Absence test kit. Seven wells tested positive for enterococci, with counts ranging from 0.1 CFU/100 mL to 6.6 CFU/100 mL (mean, 1.38 CFU/100 mL). *C. perfringens* was not detected in any well. Overall, five wells (PB-3, PB-9, PB-6B, PB-7A, and PB-12) were positive for three bacterial indicators (total coliform, *E. coli*, and enterococci), and PB-3 had the highest counts for all three indicators.

Three wells were positive for total coliphages (PB-5, PB-6B, and PB-12) and four samples were positive for F^+^-specific coliphages (PB-6A, PB-6B, PB-6C, and PB-9) when analyzed by the two-step enrichment method. One well (PB-6B) was positive for both somatic and F^+^-specific coliphages. PB-6B was also positive for all three bacterial indicators. Although both types of phage are indicators of fecal contamination, it has been suggested that the F+-specific coliphages may represent the transport and survival of the RNA human enteric viruses more adequately (International Association on Water Pollution Research and Control Study Group on Health Related Water Microbiology 1991).

**Figure 4:**
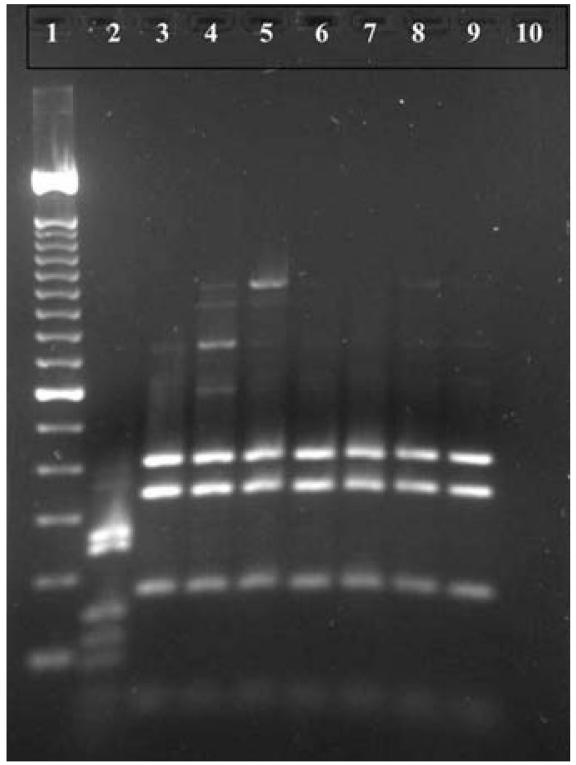
16S rDNA PCR-RFLP patterns of chromosomal extracts from bacterial isolates recovered in South Bass Island groundwater after *Dde*I digest. Lane 1, 100-bp marker; lane 2, *C. jejuni* control (ATCC no. 11168); lanes 3–9, samples isolated from South Bass Island groundwater sites PB-5, PB-12, PB-3, PB-6A, PB-6B, and PB-6C, respectively. *Dde*I restriction fragments of 421, 353, and 183 bp are indicative of *Arcobacter* as specified by Marshall et al. (1999).

A preliminary investigation of about 50% of the pure cultures isolated on the *Campylobacter*-selective media recovered *Arcobacter* spp.*,* which genetically and morphologically resembles *Campylobacter. Arcobacter* spp. were detected in seven wells (PB-3, PB-5, PB-6A, PB-6B, PB-6C, PB-9, and PB-12). The morphology of these cells was confirmed under a darkfield microscope. *Arcobacter* spp. has now been identified as an emerging cause of diarrhea in humans and is closely related to *Campylobacter* (Wesley 1997). Species-specific PCR screening for *C. jejuni* DNA from presumptive *Campylobacter* isolates was negative (Figure 4).

We detected no parasites in the 16 water samples collected. The volume of water analyzed for parasites ranged between 40 and 75 L. No cultivatable viruses were detected in the approximately 500 L of groundwater assayed. The samples were also analyzed for the presence of enteric virus DNA or RNA via PCR. Adenovirus DNA was detected at two sites, PB-9 and PB-12. The HAdV primers used were able to identify 47 HAdV serotypes, including both respiratory and enteric HAdV (Allard et al. 1992). Neither enterovirus nor norovirus was detected in any of the samples.

### Relative microbial contamination

Table 3 shows contamination of sites in the order of contamination level. PB-9, PB-12, PB-5, and PB-6B were the most contaminated, followed by PB-3, PB-6A, and PB-6C; then PB-19 and PB-7A; and PB-11 and PB-8. PB-14, PB-2, PB-4, PB-1, and PB-7B were the least contaminated. They had only some evidence of coliform and *E. coli* contamination via Colilert test. Although the contamination was widespread across the island (Figure 3), the most contaminated sites were along the southeast side of the island. The cleaner sites were all north of these, with the exception of PB-5. All (100%) of the wells were positive for both total coliform and *E. coli* by the Colilert method, but in spite of some chlorine residual, only about 65% and 47% were positive, respectively, when cultivated by membrane filtration. Enterococci and *Arcobacter* were found in 41% of the wells, whereas phage and HAdV DNA were found in 37% and 12% of the wells, respectively. Three of the five cleanest sites all carried chlorine residuals and had low turbidity. Interestingly, site PB-3, despite having chlorine residuals, was moderately contaminated; this site is located between the two most contaminated sites: PB-9 and PB-12. No chlorine residual was found in the four most contaminated sites, and PB-9 had high turbidity. PB-8 and PB-19 both produced water with high turbidities. These sites were located at the far northern tip of the island and were moderately contaminated with coliform bacteria and *E. coli*. PB-19 was also positive for enterococci.

### Hydrodynamic modeling

**Figure 5:**
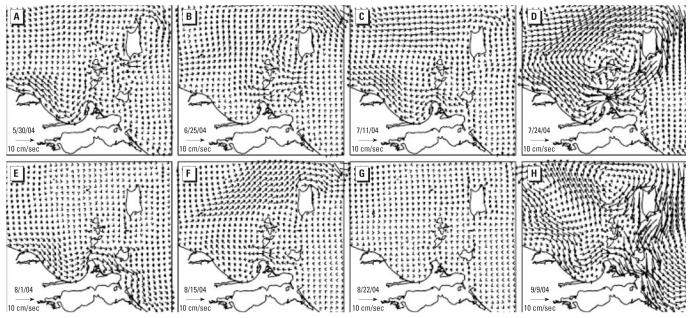
Modeled currents around South Bass Island in Lake Erie before [(*A*) 30 May, (*B*) 25 June], during [(*C*) 11 July, (*D*) 24 July, (*E*) 1 August, (*F*) 15 August, (*G*) 22 August], and after [(*H*) 9 September] the outbreak in 2004.

We examined the hydrodynamics of Lake Erie during the outbreak to explore the possible surface water–ground water interactions. Water levels forced by observed winds (O’Connor et al. 1999) and storm surges in the lake are likely to influence the subsurface aquifers. Figure 5 shows the daily averaged, vertically integrated modeled currents in the South Bass Island area plotted for 8 days during the 2004 outbreak period. On the south side of the island, currents were most commonly westward with moderated speeds ranging from 5 to 10 cm/sec (30 May, 25 June, 11 July, and 15 August 2004). There were also two examples of eastward flow (1 August and 9 September 2004), one of strong (> 20 cm/sec) westward flow (24 July 2004), and one of almost stagnant flow (22 August 2004). Currents are almost always weakly southward on the east side of the island. On the west side, currents are about evenly divided between northward and southward on these particular days. On 24 July 2004, immediately before the beginning of the largest peak in cases during the outbreak, the current pattern shows clockwise circulation around the island, with current speeds exceeding 20 cm/sec on the south shore. Water movement was almost stagnant on 22 August 2004, around the time of the sudden decrease in cases of the disease; the boil order was not initiated until 26 August 2004. The pattern of the current on 9 September 2004 before our sampling shows the strong (> 20 cm/sec) currents that can occur during fall storms.

**Figure 6:**
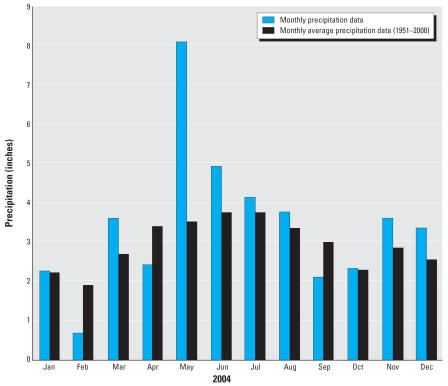
Monthly precipitation (inches) in 2004 and monthly average precipitation data (based on 1951–2000) for the North Central Climatic region of Ohio in 2004. Reproduced with permission from ODH (2005).

**Figure 7:**
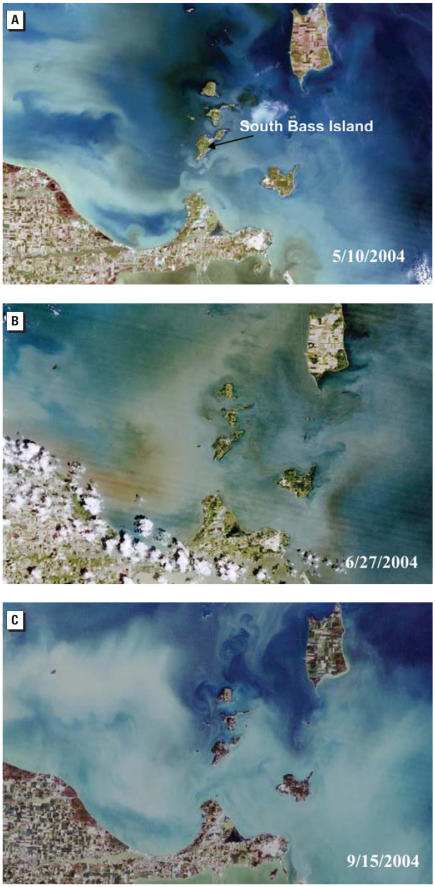
Landsat satellite images of Lake Erie around South Bass Island 10 May 2004 (*A*), 27 June 2004 (*B*), and 15 September 2004 (*C*) (OhioLINK 2004).

The monthly rainfall in 2004 was on average greater than the 50-year monthly averages for the area (Figure 6) and in fact was > 200% for May and 120% for June. Three true-color LandSat images of western Lake Erie from 10 May 2004, 27 June 2004, and 15 September 2004 are shown in Figure 7 (OhioLINK 2004). Biological activity (greenish color) is indicated on the northeast of the Island in the Figure 7A (10 May), but most of the turbidity appears to be inorganic (white and gray). By 27 June 2004 (Figure 7B), the reddish colors in the lake indicate a dramatic increase in biological activity or suspended inorganic material (clay). Finally, by 15 September 2004 (Figure 7C), the activity (or turbidity) diminished considerably except in Sandusky Bay.

## Discussion

The South Bass Island waterborne outbreak, which took place between June and August 2004, is one of the largest documented in the Great Lakes in the last decade. Our investigation indicated that the fecal contamination was massive and widespread throughout the groundwater on South Bass Island. The multitude of pathogens detected in the population, including *Camplylobacter* and norovirus, suggests that human wastes were the source of the contamination originating from wastewater facilities, septic tank effluent discharges, or possibly septage. The ODH reported that 78% and 31% of the wells were positive for total coliform and *E. coli,* respectively. About 63% of the 22 wells at depths between 0–50 feet and 18.5% of 54 wells at depths of 51–162 ft tested positive for *E. coli*. Some of the wells were sampled approximately 1 month after the outbreak during our more intensive microbial investigation (15–21 September); 100% were positive for *E. coli* in our analysis using the Colilert method, which has been known to recover chlorine-injured bacteria (McFeters et al. 1993).

*Campylobacter* was isolated from 16 stool samples and one well sample during the investigation by the CDC in late August (O’Reilly et al. 2007) but not in our well samples. In our study, seven water samples that were presumptively positive for *Campylobacter* spp. were identified as being contaminated with *Arcobacter. Arcobacter* spp. can be differentiated from other *Campylobacter*-like bacteria by two distinctive features: They grow at 15°C, and they are aerotolerant (Wesley 1997). Recent studies suggest that *Arcobacter*, especially *Arcobacter butzleri*, is associated with persistent, watery diarrhea and bacteremia in infected patients (Vandenberg 2004). Little is known about the mechanisms of pathogenicity, potential virulence factors, or clinical importance of *Arcobacter* spp. because these organisms are often misidentified as a *Campylobacter* spp. if specific testing to species level is not performed (Diergaardt et al. 2004). It is uncertain how frequently *Arcobacter* can be found in sewage, surface water, or groundwater. The high prevalence of *Arcobacter* in these water samples and the ability of the bacteria to grow at cool temperatures further support its potential as a waterborne pathogen. *Arcobacter* spp. should be considered as one of the emerging waterborne bacterial pathogens, and waters should be further monitored for this bacterium.

We did not detect *Cryptosporidium* oocysts or *Giardia* cysts in the present study using U.S. EPA method 1623 (U.S. EPA 2001c). These organisms are generally detected in surface waters and in drinking waters with inadequate filtration (Betancourt and Rose 2004). This suggests that the groundwater on South Bass Island was not under the direct influence of surface water because the presence of these parasites in water is normally the result of surface runoff (Hancock et al. 1998). The microscopic particulate analysis test run by the Ohio EPA also corroborated these results, returning a low risk of groundwater under the “direct” influence of surface waters. Although a few cases of parasitic infections were reported (three cases of giardiasis), these could have been acquired from contact with the lake. The hydraulic conductivity between the lake and the groundwater, as well as the type of glacial geology in this area, would not effectively (> 90%) remove the bacteria and viruses. Interestingly O’Reilly et al. (2007) reported no significant difference between attack rates for residents using well water or municipal water (treated Lake Erie water). Although not statistically significant, an increased OR was found by O’Reilly (2005) for cases with any contact with Lake Erie (13% of the cases and 8% of the controls showed illness, with a matched OR of 6.0; *p* = 0.1; 95% CI, 0.7–276).

Cell culture analysis showed no culturable virus; chlorination of the wells before sampling may have inactivated the viruses. Chlorinators were turned off just before collection of samples. Adenovirus DNA was detected in wells PB-9 and PB-12, which shows that these wells were vulnerable to human wastes (both wells were < 51 ft deep) and that the use of multiple cell lines may be necessary in cell culture analysis (ODH 2005). The presence of human adenoviral DNA indicated that groundwater on South Bass Island was affected by human sewage. The indicator viruses, in this case coliphages, were also detected (in 37.5% of the wells) and are highly indicative of sewage contamination. They are also useful as viral indicators of groundwater contamination because of their colloidal nature and ability to move through the subsurface.

Overall, wells PB-5 (Horny Toad), PB-6B (Island Club well 2), PB-9 (airport), and PB-12 (Skyway Lounge) were among the most contaminated sites; these wells were positive for all three bacterial indicators, coliphages, and *Arcobacter* spp. PB-9 and PB-12 were positive for adenovirus DNA. At PB-9, an on-site septic system was used for waste-water treatment; this could have been the possible source of the contamination in the well. Both PB-12 and PB-6B were connected to a privately owned treatment facility with discharge to Lake Erie.

The possible sources of human fecal wastes on this island included 149 household sewage treatment systems, 81 of which discharge to the subsurface and 68 of which discharge to Lake Erie. Also, septage was also applied to the land in the center of the western portion of the island, between Catawba and Put-in-Bay Rd.; the application was discontinued during this outbreak. On-site waste-water disposal systems, which discharge to the subsurface, have been shown to readily contaminate ground and surface waters. Virus transport in karst and porous aquifers can be rapid, ranging from 8 to 54 m/day (Paul et al. 1997). Viruses, bacteria, and parasites have been reported at concentrations of 10^2^–10^5^/L in sewage depending on the treatment scheme (type of secondary treatment) and level of disinfection (National Research Council 1998; Rose et al. 2001). The high concentrations of bacteria allowed as a part of the NPDES in sewage effluent discharges (2,000 CFU/100 mL) suggest that disinfection of the wastewater was limited (Ohio EPA 2005). The porous aquifer on the island provided little to no natural filtration that might normally occur during water movement through the soil. In addition, fractures in the limestone aquifer would have allowed for the transport of bacteria and viruses throughout the subsurface. Many existing on-site septic systems were installed in areas of thin to absent soils, whereas other wastewater systems discharge to Lake Erie (Ohio EPA 2005). Moreover, the microscopic particulate analysis test performed by the Ohio EPA on three of the island wells found no surface water indicators, suggesting that groundwater contamination through the infiltration of lake water and poorly installed sewage systems is a more likely source of contamination (Ohio EPA 2005).

Extreme precipitation events have also been shown to be related to waterborne outbreaks (Curriero et al. 2001; Morris and Levin 1995; Rose et al. 2000). Curriero et al. (2001) observed a 2-month lag statistical association between extreme rainfall events and disease outbreaks from groundwater. This is likely due to the transport time for pathogen movement from the source (i.e., sewage) to the exposure site (i.e., groundwater wells) and the time required for disease incubation and disease reporting. The average monthly precipitation in the north central region of Ohio was at a record high in late May 2004 (ODH 2005). Suspected disease cases were first noted by 30 May, followed by a small increase in June; by July (2 months later) a 4-fold increase in cases was reported. The rainfall probably contributed to a higher water table, as well as increased run-off and flushing of contaminants into the subsurface and Lake Erie.

We suggest that massive groundwater contamination on the island was likely caused by transport of microbiological contaminants from sewage discharges to the lake and to the subsurface from wastewater treatment facilities and septic tanks after extreme precipitation events in May, June, and July 2004. This may have raised the water table, saturated the sub-surface, and—along with very strong Lake Erie currents on 24 July—forced a surge in water levels and rapid surface water–groundwater interchange throughout the island. Our microbiological monitoring data showed that the highest amount of contamination was present on the southwestern side of the island. The Landsat images and the hydrodynamics of the lake at that time also show that the southwestern end of the island was affected. The amount and timing of the rainfall (2-month lag) and the strong southwestward currents on 24 July occurred directly before the beginning of the peak of disease cases, which occurred between 25 July and 17 August 2004 (Figure 2). Rapid transport and interchange likely occur between the lake and groundwater as the water levels rise as a result of storm surges. These combinations of factors and information can be used to examine vulnerabilities in other coastal systems.

Since the outbreak, the current goal has been to supply the entire island with fully treated drinking water from Lake Erie. To protect public health, routine monitoring and disinfection of groundwater for potable use on the island was made mandatory and septage disposal was discontinued. Although little attention is often given to wastewater discharges and septic drainfield effects, these issues are being addressed by the health department and the Ohio EPA; an island-wide sewer is also a goal. In spite of having fully treated and disinfected water, the multiple-barrier concept should be used for all waters. Source protection is still needed for both groundwater and surface water in order to fully protect both drinking and recreational waters.

Outbreaks represent observable epidemics and are generally considered to be under-reported. Endemic waterborne disease may still be occurring without being appropriately documented. Because of the multitude of pathogens involved and infection with bacteria such as *Campylobacter* (associated with Guillain Barré disease) (Mishu et al. 1993), the possible chronic outcomes should be followed in the population exposed in this outbreak. In addition, the role of *Arcobacter* as a waterborne pathogen should be further evaluated. The present study supports the use of remote-sensing information, climatic data, and hydrodynamic modeling to examine high-risk water contamination periods, particularly for islands and coastal systems in the Great Lakes.

## Figures and Tables

**Figure 1:**
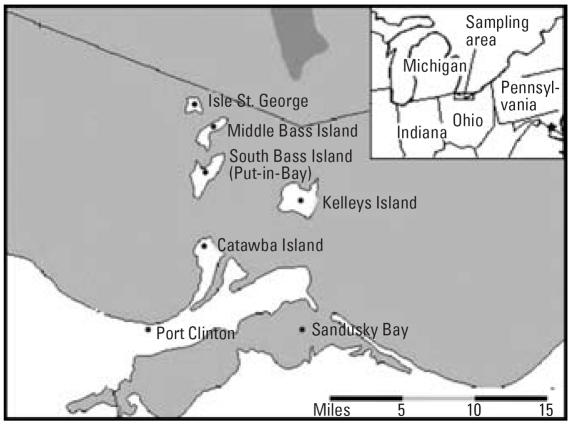
Map showing South Bass Island in Lake Erie and in relation to the State of Ohio (U.S. Geological Survey 2006).

**Table 1:** Primer sets for virus detection. The equivalent original volume of water analyzed for each sample ranged between 4.58 L and 6.46 L for NV, between 1.37 L and 1.94 L for HAdV and between 2.29 L and 3.23 L for HEntV.

Virus group, primer	Sequence (5′ to 3′)	Amplicon (bp)	Reference
NV			
NVp110	AC(A/T/G)AT(C/T)TCATCATCACCATA	398	Le Guyader et al. 1996
NVp36	ATAAAAGTTGGCATGAACA		
HAdV			
AV-A1	GCCGCAGTGGTCTTACATGCACATC		
AV-A2	CAGCACGCCGCGGATGTCAAAGT	300	Allard et al. 1992
AV-B1^a^	GCCACCGAGACGTACTTCAGCCTG		
AV-B2^a^	TTGTACGAGTACGCGGTATCCTCGCGGTC	143	Allard et al. 1992
HEntV			
ENT-up-1	GTAGATCAGGTCGATGAGTC		Fong et al. 2005
ENT-down-1	AC(T/C)GG(A/G)TGGCCAATC	330	De Leon et al. 1990
ENT-up-2^a^	CCTCCGGCCCCTGAATG		De Leon et al. 1990
ENT-down-2^a^	ATTGTCACCATAAGCAGCC	154	Fong et al. 2005

a Primers used for the second round of PCR.

**Table 2:** Physical and chemical data of groundwater collected in September 2004.

Site	Sample ID	Date collected	Free Cl_2_ (mg/L)	Total Cl_2_ (mg/L)	pH	Turbidity (NTU)	TDS (ppm)
Put-in-Bay well	PB-1	16 Sep	ND	0.04	7.12	0.08 (0.32)	410
Bird’s Nest Resort^a^	PB-2	15 Sep	ND	ND	7.2	0.1	480
Clinsters	PB-3	21 Sep	0.1	0.39	7.4	0.01	630
Fox’s Den^a^	PB-4	15 Sep	0.08	0.1	6.8	0.12	520
Horny Toad	PB-5	16 Sep	ND	ND	6.8	0 (0.37)	490
Island Club well 1	PB–6A	21 Sep	ND	ND	7	0.05	370
Island Club well 2	PB–6B	21 Sep	ND	ND	7.4	0.01	560
Island Club well 3	PB–6C	21 Sep	ND	ND	7.07	0	620
ODNR State Park well 1	PB-7A	20 Sep	ND	ND	7.11	0.05 (0.31)	510
ODNR State Park well 2	PB-7B	20 Sep	ND	ND	6.92	0.05 (0.07)	820
OSU Stone Lab	PB-8	20 Sep	ND	ND	7.1	6.48 (5.53)	1,370
Airport^a^	PB-9	15 Sep	ND	ND	6.92	4.14	500
Saunders South	PB-11	20 Sep	0.13	0.2	7.4	0.26 (0.31)	570
Skyway Lounge^a^	PB-12	16 Sep	ND	ND	7.2	0.25 (0.43)	490
Victory Park Resort^b^	PB-14	15 Sep	0.49	1	7	0.08	960
ODNR Oak Point	PB-19	16 Sep	ND	ND	7.09	3.86 (10.74)	600

ND, not detected. The turbidity of all samples was measured in the field by the Ohio EPA; the turbidity readings taken at the Michigan State University laboratory are shown in parentheses.

a Chlorinator was turned off before sampling.

b Bleach was added directly into the well by the owner for decontamination.

**Table 3:** Contamination in samples shown in order from the highest to the lowest bacterial indicator and virus counts.

	Bacteria (CFU 100/mL)						Coliphages (enrichment/L)		Enteric viruses
Sample ID	Total coliform (MF)	Total coliform (Colilert)	*E. coli* (MF)	*E. coli* (Colilert)	Enterococci	*Arcobacter*	Total	F-specific	HAdV
PB-9	7.8	+	1.3	+	1.9	+	< 1	+	+
PB-12	7.7	+	0.3	+	0.6	+	+	< 1	+
PB-6B	38	+	0.4	+	0.1	+	+	+	−
PB-5	3.4	+	< 0.1	+	2	+	+	< 1	−
PB-3	90	+	4	+	6.6	+	< 1	< 1	−
PB-6C	26	+	0.1	+	< 0.1	+	< 1	+	−
PB-6A	5.9	+	< 0.1	+	< 0.1	+	< 1	+	−
PB-19	12.8	+	< 0.1	+	5.9	< 0.1	< 1	< 1	−
PB-7A	3.7	+	0.7	+	5	< 0.1	< 1	< 1	−
PB-11	2.2	+	0.9	+	< 0.1	< 0.1	< 1	< 1	−
PB-8	3.3	+	2.6	+	< 0.1	< 0.1	< 1	< 1	−
PB-14	< 0.1	+	< 0.1	+	< 0.1	< 0.1	< 1	< 1	−
PB-2	< 0.1	+	< 0.1	+	< 0.1	< 0.1	< 1	< 1	−
PB-4	< 0.1	+	< 0.1	+	< 0.1	< 0.1	< 1	< 1	−
PB-1	< 0.1	+	< 0.1	+	< 0.1	< 0.1	< 1	< 1	−
PB-7B	< 0.1	+	< 0.1	+	< 0.1	< 0.1	< 1	< 1	−

Abbreviations: +, positive; −, negative; MF, membrane filtration. All samples were negative for *C. perfringen, Cryptosporidium* spp., *Giardia* spp., noroviruses, and human enteroviruses.
